# The multipartite mitochondrial genome of *Cynanchum auriculatum* (Gentianales: Apocynaceae)

**DOI:** 10.1080/23802359.2019.1673683

**Published:** 2019-10-04

**Authors:** Chang-Kug Kim, Yong-Kab Kim

**Affiliations:** a Genomics Division, National Institute of Agricultural Sciences, Jeonju, Korea;; b School of Electrical Information Communication Engineering, Wonkwang University, Iksan, Korea

**Keywords:** Apocynaceae family, *Cynanchum auriculatum*, mitochondrial genome

## Abstract

*Cynanchum auriculatum* is a Chinese herbal medicine species in the family Apocynaceae. The mitochondrial genome of *C. auriculatum* has heteroplasmy and consists of two chromosomes (chromosomes I and II), the lengths of which are 614,836 and 426,495 nucleotides. The multipartite mitochondrial genome encodes 57 genes, including 37 protein-coding genes, 17 transfer RNA genes, and three ribosomal RNA genes. Including 44 overlapping genes, we identified 57 genes on chromosome I and 44 genes on chromosome II. A phylogenetic tree revealed that *C. auriculatum* is most closely related to *Asclepias syriaca*.

## Introduction

*Cynanchum auriculatum* is a perennial herb that is used to treat digestive disorders in Asia. *Cynanchum auriculatum* contains many bioactive components with antioxidant, anticancer, and antidepressant activities (Liu et al. 2018). A sample of *C. auriculatum* was obtained from the National Institute of Horticultural and Herbal Science (voucher number: NIHHS2014-3, geographic coordinate: N 36°94′38ʺ, E 127°75′33ʺ) in Eumseong, Korea. Whole-genome sequencing was performed using the Illumina Miseq platform (Illumina, CA, USA). A total of 6.6 Gb of raw reads were generated. Low-quality bases (<Q20) were trimmed using the NGS QC Toolkit (Patel and Jain 2012). The screened contigs were subjected to gap-filling and error-correction procedures using the CLC Assembly Cell package (QIAGEN Bioinformatics, CA, USA). In addition, chloroplast-derived sequences of *C. auriculatum* (Jang et al. 2016) were used as a reference.

**Figure 1.**
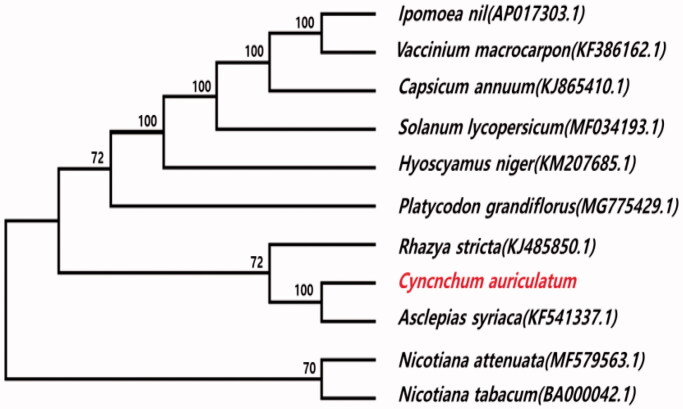
Phylogeny of *C. auriculatum* and 10 related species based on their mitochondrial genome sequences. The phylogenetic tree was constructed using the neighbor-joining method and a bootstrap test with 1000 iterations based on five common protein-coding genes from the mitochondrial genomes of 11 species.

The multipartite mitochondrial genome of *C. auriculatum* consists of the linear chromosome I (GenBank accession number: MH410146) and the circular chromosome II (MH410147). The linear nature of chromosome I was verified by an alignment of Illumina reads that showed the presence of both proximal sequences and no bridge reads with which to make a circular chromosome. We assumed that *C. auriculatum* has heteroplasmy (Woloszynska 2010) resulting from homologous recombination between the linear and circular mitochondrial chromosomes (Wei et al. 2012). The *C. auriculatum* mitochondrial chromosomes were numbered on the basis of their size. Chromosomes I and II contain 614,836 and 426,495 base pairs, respectively. The mitochondrial genome encodes 57 genes, including 37 protein-coding genes, 17 transfer RNA genes, and 3 ribosomal RNA genes. Including 44 overlapping genes, we identified 57 genes on chromosome I and 44 genes on chromosome II. In addition, we identified 17 open reading frames (ORFs) and splicing variants of the *nad1, nad2*, *nad4*, *nad5*, and *nad7* genes. The *nad5* splicing gene includes five exons in chromosome I, whereas only exons 1 and 2 exist in chromosome II. A phylogeny of *C. auriculatum* and the genomes of 10 reported Asterid species was constructed using MEGA7.0 (Kumar et al. 2016). The phylogenetic relationships were analyzed using six common protein-coding genes (*cox2, cox3, nad2, nad7, nad9,* and *rps13*). The phylogenetic tree indicated that *C. auriculatum* is closely related to *Asclepias syriaca* of the Asterids (Figure 1).

## Geolocation

The genomic DNA sample used for sequencing was obtained from the National Institute of Horticultural and Herbal Science (voucher number: NIHHS2014-3, geographic coordinate: N 36°94′38ʺ, E 127°75′33ʺ).
